# The stage of head and neck diagnosed malignancies in COVID‐19 outbreak versus before outbreak: A retrospective study

**DOI:** 10.1002/cnr2.1801

**Published:** 2023-03-16

**Authors:** Fatemeh Jahanshahi, Mahboobe Asadi, Alireza Movahedi

**Affiliations:** ^1^ Student Research Committee, Faculty of Medicine Iran University of Medical Science Tehran Iran; ^2^ Otorhinolaryngology and Head and Neck Department, Taleghani Hospital Shahid Beheshti University of Medical Sciences Tehran Iran

**Keywords:** cancer, COVID‐19, head and neck malignancy, neoplasm, TNM

## Abstract

**Background:**

Global COVID‐19 pandemic has affected cancer care systems. Recent studies show that the number of cases diagnosed with cancer has drastically decreased compared to the same period before the pandemic. Therefore, we are confronted with delayed diagnosis of critical cancers.

**Aim:**

The aim of this study is to investigate whether the stage of known cancers has been affected by delayed diagnosis and to compare the stages of head and neck cancers diagnosed during and before the pandemic.

**Methods:**

The present study was conducted on 132 patients with malignant head and neck tumors referred to the otolaryngology, head and neck cancer department of Taleghani Hospital from 2019 to 2021. The stage of cancers was compared between two groups of patients with head and neck malignancy referred to the Otolaryngology, Head and Neck Department of the Taleghani Hospital before and during the COVID‐19 outbreak.

**Results:**

The results from tumor (T), nodes (N), and metastases (M) (TNM) staging (*p*‐value = .015) and T score (value = 0.045) showed that the stage of tumor diagnosed in patients during the COVID‐19 pandemic significantly increased compared to patients diagnosed with a tumor before pandemic.

**Conclusion:**

In the present study, it was observed that the early symptoms of malignant head and neck tumors have been neglected by patients during COVID‐19 pandemic and resulted in delayed diagnosis. This result may be explained by the fear of COVID‐19 infection in patients, which discouraged them from visiting a doctor at healthcare centers.

## 1 INTRODUCTION

In December 2019, after the first reported cases of the new and unknown severe acute respiratory syndrome Coronavirus2 (SARS‐CoV‐2) in Wuhan, China, this virus spread worldwide and turned into a global outbreak. The effects of this global epidemic on healthcare systems, specifically cancer care systems, have been significant.^1^ Since patients diagnosed with cancer are more prone to be infected with this virus, with higher morbidity and mortality, it was hypothesized that the fear of getting infected discouraged cancer patients from follow‐up visits and continuing their treatments. Moreover, the involvement of most systems in the diagnosis and treatment of a large number of patients with COVID‐19 resulted in less attention to patients with cancer. Therefore, being exposed to the COVID‐19 virus during the use of diagnosis modalities, limited capacities for elective surgeries, and patients' reluctance to follow up on their treatment after a definite diagnosis might lead to a delay in either diagnosis or treatment of important cancers.^2^, ^3^, ^4^, ^5^

Different countries implemented various approaches to resolve this issue. One of these approaches is telemedical services to recognize probable cancer red flags rather than postponing the investigation of unlikely symptoms. However, the diagnosis and treatment of cancers are not back to the norm before the pandemic.^6^, ^7^

In 2020, De Vincentiis et al.^8^ have shown that the rate of cancer diagnosis has decreased by 39% compared to 2018 and 2019. In these years, the COVID‐19 pandemic has been a significant factor and has adversely affected the diagnosis and screening of cancers. However, the role of both patients and healthcare systems in creating this problem needs further investigation.

In this work, the potential effects of COVID‐19 outbreak on the stage of cancers diagnosed during the pandemic were studied.

## 2 METHODS

### 2.1 Study design and sampling

The present study was a retrospective study conducted on 132 patients with malignant head and neck tumors referred to the otolaryngology, head and neck department of Taleghani Hospital from 2019 to 2021. Information of the patient has not been revealed in the study findings. The stage of cancer was compared between two groups of patients from before and during the COVID‐19 outbreak. All enrolled patients were diagnosed with malignant head and neck tumors—confirmed by postoperative or biopsy pathology reports. In this study, in addition to reviewing the clinico‐pathological TNM staging by a specialist for enrolling the patients, gender and age of the two groups were considered. The known cancer cases with recurrence malignancy were excluded from the study. At the end, the patients were asked two questions: (1) Is there any delay between the onset of the symptoms and visiting a doctor? (2) What is the reason for this delay? Finally, the information was then recorded.

### 2.2 Statistical analysis

After collecting the data, the Kolmogorov–Smirnov test showed normal distribution of the data. Therefore, parametric statistical tests were used. The quantitative variables were compared using descriptive statistics (average). The qualitative variables were assessed using the chi‐square test. Statistical analysis was performed using SPSS software Version 22. The statistical significance was considered to be less than .05.

## 3 RESULTS

In this study, 125 new cases of head and neck neoplasm (61 males and 64 females with a mean age of 53.72) were referred to otolaryngology, head and neck cancer department of Taleghani Hospital, between 21 January 2020 and 20 January 2021. The clinical characteristics of head and neck neoplasms in the patients before and during the COVID‐19 pandemic have been shown in Table 1. Based on the pathology records, 50 patients (40%) had a benign tumor, and the remaining 75 patients (60%) reported malignant neoplasms. Among the malignant group, 68 patients (90.6%) were with locally advanced diseases undergoing definitive treatment, four patients (5.3%) had treatment discontinuation (two adjuvant radiotherapies and two surgeries), and three (4%) were with metastatic diseases undergoing systemic therapy. Among the malignant neoplasm, thyroid carcinoma (papillary thyroid carcinoma) was the most common pathology, followed by laryngeal squamous cell carcinoma. Besides, pleomorphic adenoma was the most common pathology in the benign group. Moreover, three cases of SARS‐Cov‐2 infections were confirmed during hospitalization in the malignant group.

**TABLE 1:** The clinical characteristics of head and neck neoplasms before and after the covid 19 pandemic.

	Before covid (21 January 2019 to 20 January 2020)	During covid (21 January 2020 to 20 January 2021)
Gender	66 males	61 males
	72 females	64 females
Age	Mean: 49.28	Mean: 53.72
Benign	58.69%	40%
Malignant	41.30%	60%
	Thyroid = 15	Thyroid = 16
	Salivary glands = 12	Salivary glands = 9
	Larynx = 10	Larynx = 12
	Tongue = 6	Tongue = 8scc
	Others = 14	Others = 30
Complication	Hematoma = 1	Hematoma = 1
	Seroma = 1	Wound dehiscence = 1
		Covid‐19 infection = 3

On the other hand, during the study time, 138 new head and neck neoplasms were documented in our medical records. Among these, 57 patients (41.3%) were assigned as a malignant neoplasm, undergoing definitive treatment. No metastatic diseases were reported.

We compared the clinic‐pathological TNM stage of the patients, to perceive the impact of medical care suspension due to the COVID‐19 pandemic on the head and neck cancer stage, (Table 2). Comparing the cancer stage—based on the TNM stage—revealed a significant increase in tumor T‐score and accordingly tumor stage, due to the pandemic (*p*‐value: .045, .015); while no remarkable impact was detected in lymph node involvement and metastatic status (*p*‐value: .479, .423).

**TABLE 2:** The clinical TNM staging of head and neck solid cancers before and after the covid 19 pandemic.

	Before covid‐19 (21 January 2019 to 20 January 2020)		During covid‐19 (21 January 2020 to 20 January 2021)		*p*‐value
TNM stage	Stage 1	27	Stage 1	15	.015
	Stage 2	21	Stage 2	24	
	Stage 3	9	Stage 3	33	
	Stage 4	0	Stage 4	3	
T (size and extension)	T 1	27	T 1	15	
	T 2	18	T 2	20	
	T 3	9	T 3	36	
	T 4	3	T 4	4	
N (node)	N 0	33	N 0	66	.479
	N 1	21	N 1	9	
	N 2	3	N 2	0	
	N 3	0	N 3	0	
M (metastasis)	M 0	57	M 0	72	.423
	M 1	0	M 1	3	

## 4 DISCUSSION

During the COVID‐19 pandemic, cancer management has been affected at multiple levels. Since patients tended to stay at home during the pandemic and the National Health Services rapidly switched their comprehensive health care focus to COVID‐19 patients, cancer screening processes were either suspended or slowed down. These factors have caused a delay in cancer diagnosis through screening processes and resulted in identifying cancer tumors at more advanced stages associated with more complications.^9^, ^10^, ^11^, ^12^ In addition, the secondary care in healthcare systems experienced dramatic changes. When possible, surgeons and oncologists modified their treatment protocols to minimize hospitalization and preserve healthcare capabilities for COVID‐19 patients.^13^ The specific objective of this study is to investigate the potential impacts of COVID‐19 pandemic on the stage of head and neck cancers at the time of diagnosis in patients who were diagnosed through experiencing symptoms and seeking medical attention.

For most head and neck solid cancers, the TNM staging system is a prevalent method to describe the extent of the tumor. This study suggests that the COVID‐19 pandemic can lead to progression in the stage of head and neck cancers at the time of diagnosis. Although this progression can happen to all cancers, the overall impact of the COVID‐19 pandemic on the stage of cancers might vary in different types of cancer.

COVID‐19 pandemic can affect the TNM stage of cancers in different ways. The analysis of this study reveals that there is a substantial rise in T‐stage for most head and neck cancers. However, the adverse changes in the status of nodal involvement and cancer metastasis are not significant. The results of conducted interviews showed that among the 75 patients diagnosed before the COVID‐19 pandemic, only 12 patients (16%) had a delay in seeing a doctor due to reasons such as being unaware of the importance of symptoms or not having access to medical centers. However, among the 57 patients diagnosed during the COVID‐19 pandemic, 41 individuals (71.9%) stated that they ignored the symptoms for a long time. In addition, 38 of these patients (92.6%) mentioned fear of getting infected with COVID‐19 at healthcare centers as their main reason for being reluctant to seek medical attention. Only 3 of these 41 mentioned reasons were related to reasons other than fear of being infected by COVID‐19 infection in the hospital.

The current study was conducted at a tertiary care center; thus the collected data is limited to patients who were referred to the center. In addition, our data did not capture the brunt of cancer upstaging on cancer‐specific survival. However, several studies have reported the adverse effects of COVID‐19 on cancer survival. Sud et al.^14^ estimated the hazard ratio for diagnostic delays in the 20 most common cancers (including esophagus, oral cavity, oropharynx, and thyroid). They suggested that treatment suspension of 2–6 months will lead to progression in early‐stage tumors and turn a curable cancer to incurable one. They also showed that even small delays in diagnosis can lead to crucial survival decrements in young patients and adults aged ≥70 years. In another study by Maringe et al.^15^ the impact of diagnosis delay on cancer survival was estimated in breast, colorectal, esophageal, and lung cancers. Their results indicated that diagnostic delay as a result of the COVID‐19 pandemic led to a remarkable increase in cancer deaths in the UK. They estimated between 3291 and 3621 avoidable deaths across the four major cancer types with a total additional year of life lost (YLLs) 59 204–63 229 years.

This study investigated the impact of COVID‐19 pandemic on diagnosis and treatment of cancers by considering other causes both before and during the corona were sustained.^16^, ^17^, ^18^

In this study, the data was obtained only from patients in Iran, however, since there is a uniform cancer stage system across most countries, this prediction of head and neck cancer stage transformation during COVID‐19 is potentially applicable to other countries, especially in areas where population structure and cancer prevalence are generally similar to Iran. It is worth mentioning that the cancer stage transformation during the pandemic is a variable target because of the fluctuating change in COVID‐19 cases. Additionally, estimating the adverse effect of severe acute respiratory syndrome coronavirus 2 (SARS‐CoV‐2) infections on head and neck cancer stage might be more challenging than other sites of cancers because it contains high concerns of the disease spread for both patients and healthcare providers.

Although the information on cancer upstaging due to the pandemic effect is of paramount clinical importance, data on the transformation of the cancer stage caused by the pandemic are scarce. The present study is the first documented study focused on the head and neck cancers stage with considering the TNM staging,^19^ however, it is associated with a few limitations.

The main limitation of this study is that it only focuses on solid head and neck invasive cancers in adult population, further studies should be conducted on cancers of childhood, non‐solid cancer, and noninvasive and pre‐cancerous lesions (carcinoma in situ, metaplasia, and dysplasia). Moreover, substantial heterogeneity remains in cancer pathologic types across the different head and neck cancers.

Additionally, this study is a single‐center study, therefore, this data might not fully represent the COVID‐19 pandemic effect.

Lastly, the present study did not consider any data on the socioeconomic status of the patients. It would be valuable to know how the socioeconomic status of the patients would change and how these alterations influenced their treatment plans.^20^

## 5 CONCLUSION

Notwithstanding the mentioned limitations, the study suggests that during the COVID‐19 pandemic, the fear of coronavirus infection discouraged people to pay attention to and follow up on the head and neck cancer red flags, likely due to the lack of knowledge about how serious these red flags are. On the other hand, the health care systems were more focused on COVID‐19 patients. Although, problems such as lack of access of patients to medical services, economic problems, and unawareness have always interfered with the diagnosis and treatment of cancers, but during the COVID‐19 pandemic, the fear of infecting with COVID‐19 has also been added to these factors.

While researchers should focus on avoidable factors affecting the treatment plan and facilitate alternative strategies to assist cancer patients, these findings suggest several courses of action including cancer awareness, regular screening and checkups, and telemedicine cancer care to minimize the effect of the COVID‐19 pandemic on cancer management systems. Moreover, any healthcare suspension should be avoided, and cancer screening and staging techniques should be more concentrated, especially at the tumor T stage, to preserve the therapeutic resources. This might be achievable by an extension in technical supplies for endoscopy and different imaging modalities and increasing the health care staff.

## AUTHOR CONTRIBUTIONS

**Fatemeh Jahanshahi:** Conceptualization (equal); data curation (equal); methodology (equal); resources (equal); writing – original draft (equal); writing – review and editing (equal). **Mahboobe Asadi:** Conceptualization (equal); software (equal); supervision (equal); writing – review and editing (equal). **Alireza Movahedi:** Formal analysis (equal); investigation (equal); methodology (equal); software (equal); writing – original draft (equal); writing – review and editing (equal).

## CONFLICT OF INTEREST STATEMENT

The authors have stated explicitly that there are no conflicts of interest in connection with this article.

## ETHICS STATEMENT

The present study is in compliance with ethical standards and standards of research involving humans. This article does not contain any studies involving animals performed by any of the authors. In our institute at the time of the study, retrospective studies that were just medical field reading and without medical intervention were not presented to the ethics committee and therefore were not assigned an ethical code to them, but there was a rule to which information of the patient should not be revealed in the study findings.

## CONSENT TO PARTICIPANTS

Written informed consent was obtained from the patients for participating in the study and publication of the results retrieved form their information before enrolling stage. Information of the patient have not been revealed in the study findings.

## Data Availability

Data in the current study are available from the corresponding author on reasonable request.
